# Surgical instrument-tissue interaction recognition with multi-task-attention video transformer

**DOI:** 10.1007/s11548-025-03546-3

**Published:** 2025-11-11

**Authors:** Lennart Maack, Berk Cam, Sarah Latus, Tobias Maurer, Alexander Schlaefer

**Affiliations:** 1 Institute of Medical Technology and Intelligent Systems, Hamburg University of Technology, Hamburg, Germany; 2 Martini-Klinik Prostate Cancer Center, University Medical Center Hamburg-Eppendorf, Hamburg, Germany; 3 Department of Urology, University Medical Center Hamburg-Eppendorf, Hamburg, Germany

**Keywords:** Deep learning, Video transformer, Surgical triplet recognition, Surgical activity recognition

## Abstract

****Purpose**:**

The recognition of surgical instrument-tissue interactions can enhance the surgical workflow analysis, improve automated safety systems and enable skill assessment in minimally invasive surgery. However, current deep learning methods for surgical instrument-tissue interaction recognition often rely on static images or coarse temporal sampling, limiting their ability to capture rapid surgical dynamics. Therefore, this study systematically investigates the impact of incorporating fine-grained temporal context into deep learning models for interaction recognition.

****Methods**:**

We conduct extensive experiments with multiple curated video-based datasets to investigate the influence of fine-grained temporal context for the task of instrument-tissue interaction recognition using video transformer with spatio-temporal feature extraction capabilities. Additionally, we propose a multi-task-attention module that utilizes cross-attention and a gating mechanism to improve communication between the subtasks of identifying the surgical instrument, atomic action, and anatomical target.

****Results**:**

Our study demonstrates the benefit of utilizing the fine-grained temporal context for recognition of instrument-tissue interactions, with an optimal sampling rate of 6-8 Hz identified for the examined datasets. Furthermore, our proposed MTAM significantly outperforms state-of-the-art multi-task video transformer on the CholecT45-Vid and GraSP-Vid datasets, achieving relative increases of $4.8 \%$ and $5.9 \%$ in surgical instrument-tissue interaction recognition, respectively.

****Conclusions**:**

In this work, we demonstrate the benefits of using a fine-grained temporal context rather than static images or coarse temporal context for the task of surgical instrument-tissue interaction recognition. We also show that leveraging cross-attention with spatio-temporal features from various subtasks leads to improved surgical instrument-tissue interaction recognition performance. The project is available at: https://lennart-maack.github.io/InstrTissRec-MTAM.

## Introduction

Minimally invasive surgery (MIS) is increasingly being adopted due to its benefits of reduced postoperative pain, shorter hospital stays, and faster patient recovery compared to open surgery [19]. Despite these advantages, preventable medical errors and intraoperative complications remain significant concerns, highlighting the ongoing need for strategies to enhance surgical safety and efficiency [3].

The identification of intraoperative safety risks and areas of improvements can translate to fewer complications and shorter hospital stays, ultimately leading to better patient outcomes [2]. Advances in surgical computer vision enable automated analysis of surgical procedures, providing intraoperative context-aware support for surgeons and enhancing postoperative surgical archives and education [14].

The majority of pioneering work in the field of surgical computer vision has concentrated on developing methods to recognize phases from endoscopic video [4, 17, 23]. Further research focuses on understanding fine-grained surgical scenes by tackling tasks such as instrument-, or anatomy segmentation within the surgical field using convolutional or transformer-based architectures [9, 13, 20, 22]. To describe surgical activities at a fine-grained level, for instance over a few seconds, recent research has examined the recognition and localization of instrument-tissue interactions [1, 15, 16, 24]. Such fine-grained modeling enhances workflow analysis, enables the development of automated surgical safety systems and provides a basis for detailed surgical skill assessment [14]. The accurate recognition of fine-grained instrument-tissue interactions is a challenging task due to the high visual similarity between different instrument-tissue interactions, the rapid movement of instruments and the high imbalanced class distribution among available datasets.

In order to address the aforementioned issues, recent works have proposed deep learning methods based on convolutional neural networks, multi-task learning and mixed attention techniques to integrate subtask knowledge [15, 16]. Further work utilizes knowledge distillation or contrastive learning methods to facilitate tail class recognition [5, 6, 26]. Pei et al. [18] proposed a semi-supervised framework that utilizes pseudo localization and activity labels to optimize instrument-tissue interaction recognition and localization. To integrate temporal context into the recognition task, recent work exploit temporal modeling, e.g., based on late fusion, to improve instrument tissue interaction recognition [21].

Despite the impressive results, there are limitations to the aforementioned work. First, only a very coarse temporal context, i.e., one frame per second, is used for the temporal modeling. Second, decoupled spatio-temporal extraction might be insufficient for aggregating features for fine-grained surgical activities.

In this work, we demonstrate that a fine-grained context is more advantageous for the accurate recognition of surgical instrument-tissue interactions. To determine the optimal temporal context for the task of instrument-tissue interaction recognition, we leverage publicly available datasets, previously processed only as single images or with coarse temporal context, into video-based datasets with finer temporal context and conduct systematic ablation studies. By employing state-of-the-art video vision transformer with unified spatio-temporal feature extraction capabilities, we demonstrate superiority over image-based models and decoupled spatio-temporal modeling approaches.

Another contribution in this work comprises a bidirectional Multi-Task-Attention Module (MTAM) that extends established mixed-attention approaches for instrument-tissue interaction recognition [16]. This is achieved by directly utilizing spatio-temporal features and bidirectionally leveraging representations from all subtasks via self- and cross-attention. Furthermore, a gating mechanism is employed to dynamically adjust the influence of cross-task information on each subtask representation. This way the communication between the different subtasks is enhanced, yielding coherent individual subtask and overall instrument-tissue interaction recognition while preserving contextual consistency across the instrument–action–target relationship space.

In summary, our contributions are as follows:

- We demonstrate the advantage of leveraging the fine-grained temporal context for the task of instrument-tissue interaction recognition and determine the optimal temporal context. For this purpose, we leverage publicly available datasets, previously available only as single images, into video-based dataset and make them publicly available.
- We demonstrate superiority of video vision transformer over image-based models and decoupled spatio-temporal modeling approaches for the task of instrument-tissue interaction recognition.
- We propose a Multi-Task-Attention Module that leverages cross-attention to enhance the communication between the different subtasks. Our proposed framework outperforms the state-of-the-art multi-task video transformer on the datasets CholecT45-Vid and GraSP-Vid with a relative increase of $4.8 \%$ and $5.9 \%$, respectively.

## Materials and methods

In this section, we detail the creation of the surgical video datasets utilized in this study for instrument-tissue interaction recognition. Additionally, we describe methods for Surgical Instrument-Tissue Interaction Recognition as well as our proposed MTAM.

### Datasets

**CholecT45-Vid**: The CholecT45 dataset, a subset of Cholec80, provides multilabel triplet annotations of the form $\langle instrument, action, target \rangle$ for laparoscopic cholecystectomy videos [15, 16]. However, its sampling rate of one frame per second (FPS) results in significant temporal sparsity, possibly failing to capture transient but critical surgical actions like ’coagulate’ or ’cut’.

To address this limitation, we create CholecT45-Vid, a derivative dataset with increased label density suitable for training video-based models. We leverage the existing sparse annotations by extrapolating them to the original high-framerate videos of Cholec80. First, we identify continuous interaction events in the CholecT45 labels. We then filter for events with a duration of three to 20 s. For each event, we extract the corresponding video segment from Cholec80 at its original 25 FPS resolution. The start of the clip corresponds to the timestamp of the first frame of the event, and the end is defined by the start of the next, different triplet interaction event. We assume the triplet labels for a given second are valid for all 25 frames within that second. This protocol results in 3,634 video clips with clip durations from three to 20 s and an average duration of seven seconds. CholecT45-Vid contains 55 triplet classes, six instrument classes, 10 action classes, and 14 target classes.

**GraSP-Vid:** The original GraSP dataset consists of untrimmed surgical videos from 13 patients undergoing robotic-assisted radical prostatectomy [1]. Its surgical activity doublet annotations $\langle instrument, action \rangle$ are sparse, tied to keyframes sampled only every 35 s. To address the limitation of missing temporal context, we convert these sparse, static annotations into a densely labeled video clip dataset (GraSP-Vid) that captures motion context. Our generation protocol leverages the original annotation process, in which labels were derived from three-second video segments centered on each keyframe. We therefore extract these exact 3-second segments at 25 FPS and assign the keyframe’s label annotation to all frames within the clip. This procedure yields GraSP-Vid, a dataset of 3,080 densely labeled 3-second video clips, suitable for training video-based models. GraSP-Vid contains 49 activity doublet classes, seven instrument classes and 22 action classes.

**SAR-RARP-Vid:** The SAR-RARP dataset contains 50 untrimmed videos of suturing gestures during robotic-assisted radical prostatectomy, containing segments of eight fine-grained surgical gestures [24]. We created SAR-RARP-Vid, a dataset of isolated gesture video-clips. To generate the clips, we used the start and end timestamps provided in the original annotations to trim each segment from the source videos. Each resulting clip is labeled with the single, corresponding gesture. This procedure leads to 2,012 clips with a mean duration of six seconds.

**Fig. 1:**
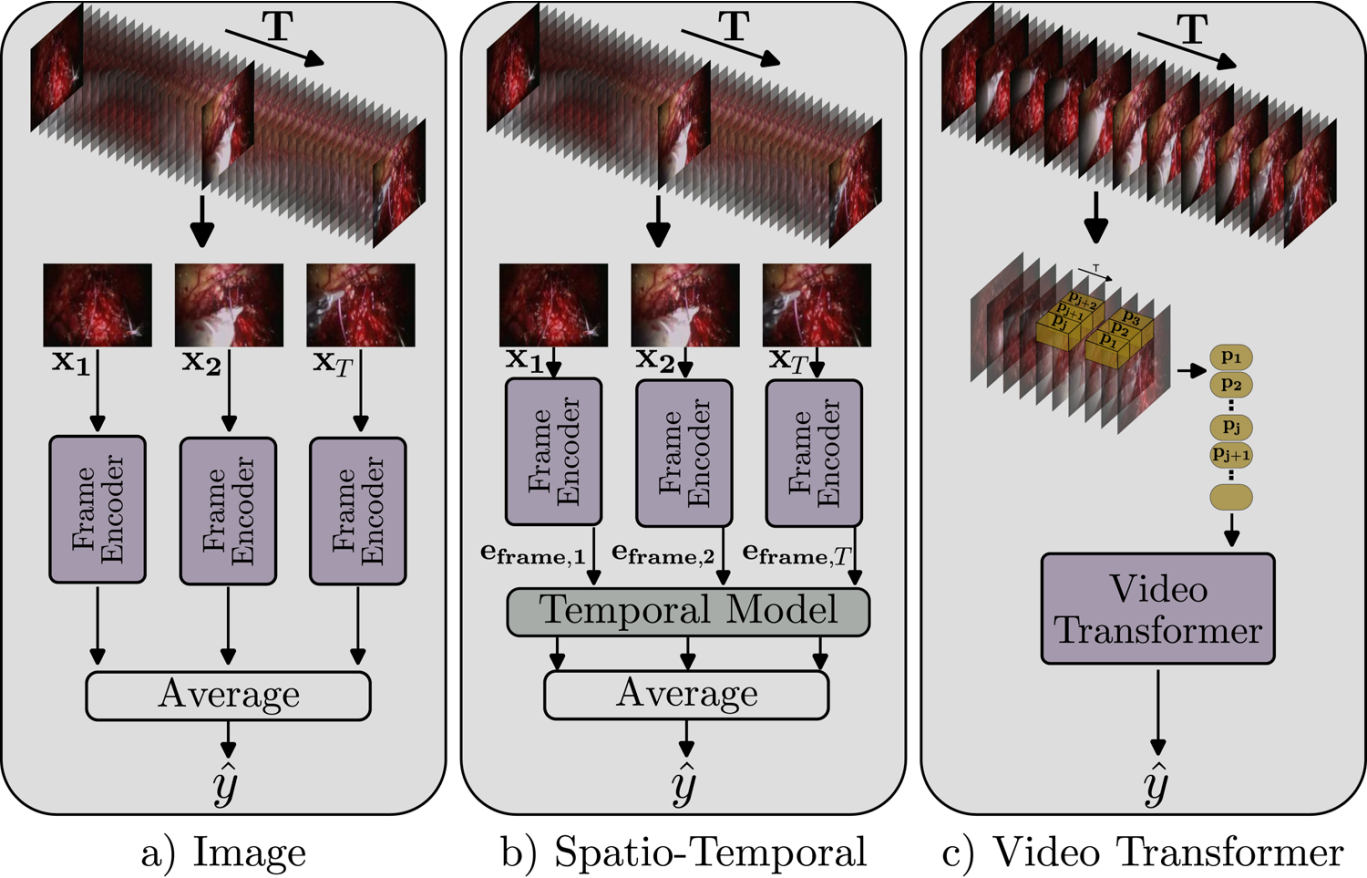
Visualization of the processing of an instrument-tissue interaction video clip using three distinct approaches: Image-based (**a)**), Spatio-Temporal (**b)**), and Video Transformer (**c)**). Approaches **a)** and **b)** utilize a lower sampling rate, while approach **c)** processes at a higher sampling rate, thereby incorporating richer contextual information

**Fig. 2:**
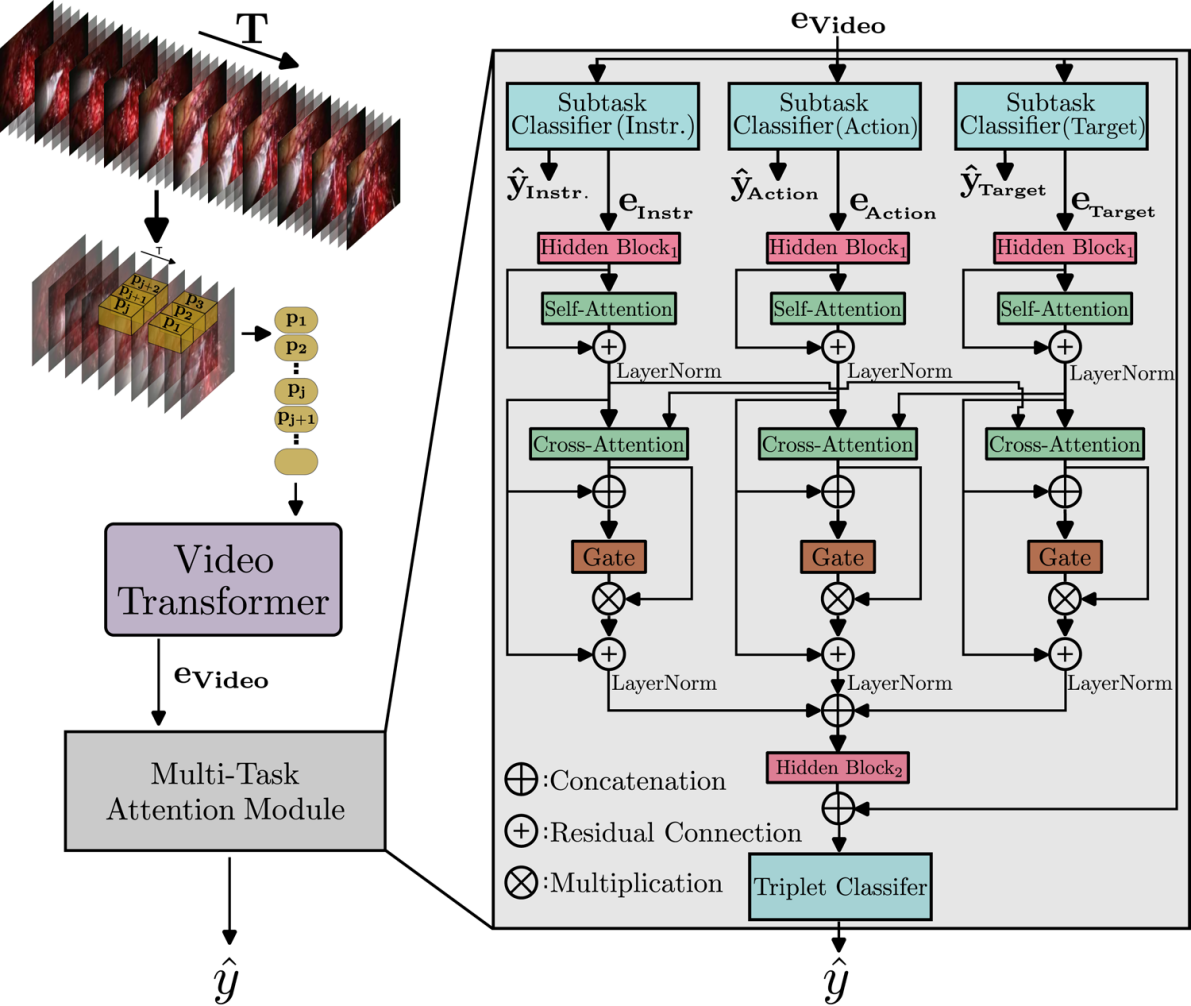
Architecture of our proposed Multi-Task-Attention Module exemplified for the triplet recognition task in the CholecT45-Vid dataset

### Methods for surgical instrument-tissue interaction recognition

In this work, we demonstrate the advantage of leveraging fine-grained temporal context for the task of instrument-tissue interaction recognition and the superiority of video vision transformer over image-based models and decoupled spatio-temporal modeling approaches.

Figure 1 illustrates approaches for processing a video clip of an instrument-tissue interaction, including state-of-the-art approaches such as image-based or spatio-temporal approaches. For input, frames are sampled from a video clip at a rate specified in hertz (Hz). Each training sample is denoted as $\{X, y \} = \{x_t, y\}_{t=1}^T$. *X* denotes a video clip of *T* frames and $x_t \in \mathbb {R}^{H \times W \times 3}$ is a RGB frame. In this work, we use datasets with single-label (SAR-RARP-Vid) and multi-label ground truth (CholecT45-Vid, GraSP-Vid). The ground truth label *y* can therefore be denoted accordingly. For the single-label case, $y \in { \{0, C\}}$ represents the ground-truth label, where *C* indicates the number of classes. For the multi-label case, *y* is denoted as $y = \{y_{c} \in \{0, 1\} \}_{c=1}^C$, with $y_{c}$ being the binary indicator telling whether the specific class ’c’ is present. For the single-label case the model is optimized with the cross-entropy loss. For the multi-label case the model is optimized with the binary cross-entropy loss averaged across all *C* classes.

**Table 1:** Performance metrics in $\%$ for different datasets. We report mean and standard deviation over five splits for CholecT45-Vid, and over three seeds for GraSP-Vid and SAR-RARP-Vid. Note: Our proposed MTAM is not applicable to single-label datasets (SAR-RARP-Vid), while the Rendevouz and MT4MTL-KD models are not applicable to doublet (GraSP-Vid) or single-label datasets; all of these instances are indicated by an X. Best scores are depicted in bold

		mAP ($\uparrow$)						Acc. ($\uparrow$)
	CholecT45-Vid				GraSP-Vid			SAR-RARP-Vid
**Model**	Triplet	Instr	Action	Targ	Doublet	Instr	Action	
Res50 [7]	23.9±2.91	85.9	59.0	42.2	20.1±1.02	86.9	18.1	61.1±4.38
SwinS [11]	30.0±0.74	93.7	64.7	48.0	22.6±0.71	90.0	21.9	68.5±2.11
Rendevouz [16]	17.5±0.22	85.2	56.1	34.3	X	X	X	X
MT4MTL-KD [6] (SwinL$\xrightarrow {/phanthom{i}}$Res18)	28.3±0.20	92.4	64.0	46.4	X	X	X	X
Res50+LSTM	23.8±1.88	90.1	59.7	40.9	20.1±0.51	88.8	17.7	65.7±0.96
Res50+Transf	27.1±2.76	93.2	63.0	44.5	22.5± 0.91	89.9	19.1	68.2±0.86
SwinS+LSTM	29.6±1.60	95.2	63.8	45.8	23.1±0.91	89.9	20.8	69.3±0.98
SwinS+Transf	29.5±3.69	96.2	64.5	48.2	24.9± 0.98	92.6	22.5	69.2±0.92
Swin3D-S [12]	33.3±1.39	**97**.**1**	67.8	52.1	30.6±0.87	92.6	26.2	80.0±0.87
MViTv2 [10]	33.8±2.02	95.4	**75**.**0**	52.9	31.2±0.44	91.6	27.2	**85.9±3.12**
MTAM (Ours)	**34.9±1.17**	97.0	69.1	**53**.**6**	**32.4±2.35**	**92**.**9**	**28**.**8**	X

**Table 2:** Computational profile of the evaluated architectures, benchmarked on a single NVIDIA RTX 3090. We report FLOPS, trainable parameters and inference latency measured using a 3x224$\times$224 input. Video and image-temporal use 16-frame clips; image models are reported per-frame (per-clip equivalent in parentheses)

Model	FLOPS ($\downarrow$)	Parameters ($\downarrow$)	Latency ($\downarrow$)	mAP-Score ($\uparrow$)
Res50	4.1G	23.7M	3.7ms(11.6ms)	23.9
SwinS	8.8G	48.9M	12.6ms(34.8ms)	30.0
Rendevouz	2.1G	14.8M	13.8ms(13.9ms)	17.5
MT4MTL-KD (SwinL$\xrightarrow {/phanthom{i}}$Res18)	**1.8G**	**11.7M**	**1.9ms(4.3ms)**	28.3
Res50+LSTM	65.8G	44.7M	12.8ms	23.8
Res50+Transf	66.2G	51.1M	12.0ms	27.1
SwinS+LSTM	140.3G	51.9M	35.3ms	29.6
SwinS+Transf	140.4G	52.8M	34.5ms	29.5
Swin3D-S	83.1G	49.6M	30.8ms	33.3
MViTv2	64.5G	34.3M	23.1ms	33.8
MTAM (Ours)	83.1G	52.4M	30.9ms	**34**.**9**

In the image-based approach (Fig. 1a)), each frame $x_t$ is processed independently by a frame encoder. The resulting frame-level predictions are then averaged across all frames in the sequence to produce a single prediction, $\hat{y}$. For the spatio-temporal approach (Fig. 1b)), each frame $x_t$ is first passed through a frame encoder to generate an embedding, $e_{frame, t}$. This sequence of embeddings is then fed into a temporal model. Finally, the outputs from the temporal model are averaged over the time dimension to yield the prediction $\hat{y}$. Figure 1c) illustrates the processing of a video clip with a more a fine-grained temporal context. A video transformer allows for unified spatio-temporal feature extraction by considering each 3D patch $p_j$ as input.

As we additionally introduce a Multi-Task-Attention Module (MTAM) in this work, the video transformer is used as a feature extractor to generate the video embedding $e_{Video}$, as depicted in Figure 2. The video embedding is further used as input for the MTAM.

### Multi-task-attention module

The Multitask Attention Module (MTAM), shown in Figure 2, is composed of subtask classifiers, self-attention, cross-attention, and linear layers. Its purpose is to improve the recognition of both individual subtasks and the overall instrument-tissue interaction by facilitating communication between them.

The video embedding, $e_{Video}$ is processed by *S* independent subtask classifiers, each designed as a single fully-connected linear layer. These classifiers serve as lightweight prediction heads, mapping $e_{Video}$ to task-specific class logits, $\hat{y}_{subtask}$, which enables efficient multi-task learning. The class logits for each subtask are jointly optimized using the binary cross-entropy loss $\mathcal {L}_{\text {BCE, i}}$ for each subtask *i*.

Subtask feature embeddings, $e_{subtask}$, are first projected to a common dimension by $\text {Hidden Block}_1$. A Self-Attention layer then captures internal relationships within each subtask’s feature embeddings. Subsequently, a bidirectional Cross-Attention layer facilitates mutual information exchange between two subtasks. After a concatenation of the inputs (subtask-specific knowledge) and outputs of the Cross-Attention Layer (mutual subtask knowledge), a gate layer is applied. The output of the Cross-Attention layer is concatenated with its input and then fed through a gate layer (a linear layer with a sigmoid function). This gating mechanism dynamically adjusts the influence of cross-task information on each subtask representation. Residual layers are also included to enhance feature refinement and stabilize gradient flow. After concatenating all subtask feature embeddings, $\text {Hidden Block}_2$ projects the combined features to a common dimension. These projected features are then concatenated with the video embedding, $e_{Video}$, and input to a classifier that predicts the final logits, $\hat{y}$. The final objective function optimized during training is defined as:

1 $$\begin{aligned} \mathcal {L} = \mathcal {L}_{\text {BCE}} + 0.5 \cdot \sum _{\text {i}=1}^{\text {S}} \mathcal {L}_{\text {BCE, i}} \end{aligned}$$

## Experiments

This section outlines the experiments conducted to evaluate our proposed methods. We detail the implementation (model architectures, setup, and hyperparameters), followed by a description of the evaluation metrics. We then present the main experimental results and conclude with an ablation study on video sampling and the impact of the MTAM components.

### Implementation details

In this study, we use the Pytorch framework and conduct our experiments on four NVIDIA V100 32GB GPUs. The input video frames are resized to 256 $\times$ 448 (H, W). For spatio-temporal and video-based models, clips are sampled to a fixed length of 16 frames. The sampling rate (Hz) is determined on a per-dataset basis. This rate is constrained by the duration of the shortest video in each dataset, as sampling a fixed number of frames requires a minimum video duration. The selected rate represents the maximum value that ensures the 16 frames can be extracted from every video within a given dataset. For the results reported in Table 1, the rates are set to 6 Hz for the CholecT45-Vid and GraSP-Vid datasets, and 8.5 Hz for SAR-RARP-Vid. During training, for videos exceeding the minimum required duration, a single 16-frame subclip is sampled from a random temporal location. For image-based models, frames are sampled from the video clips at one frame per second and used individually for training.

The selected architectures for comparison include image-based, spatio-temporal, and video transformer models. The image-based models are ResNet50 (Res50) and Swin Transformer-S (Swin-S). The spatio-temporal models utilize either an LSTM or a Transformer as the temporal component over a backbone feature extractor [8, 25]. The video transformer models are Multiscale Vision Transformers (MViTv2) and the Video Swin Transformer-S (Swin3D-S) [10, 12]. Furthermore, we compare our proposed approach with state-of-the-art methods for triplet instrument-tissue interaction recognition, including Rendevouz and MT4MTL-KD (SwinL$\xrightarrow {/phanthom{i}}$Res18) [6, 16]. Both methods are based on a ResNet18 backbone. For the CholecT45-Vid and GraSP-Vid datasets, all architectures are trained with multi-task learning. Our proposed MTAM uses a Swin3D-S as its video backbone.

Image models and video transformer models are initialized with pretrained weights from IMAGENET1K V1 and KINETICS400 V1, respectively. The weights of the MTAM and the temporal models are initialized randomly. We use a learning rate of 0.0001 for all models, determined via a hyperparameter search. The MTAM is trained with a learning rate 10 times higher than that of the video backbone to facilitate faster convergence. We apply different light data augmentations (color, brightness, contrast, blurring and defocusing). Training is conducted over 80 epochs with a batch size of 8. We employ a OneCycleLR learning rate scheduler with a cosine annealing strategy.

The code and video datasets for Surgical Instrument-Tissue Interaction Recognition will be made publicly available upon acceptance (github.com/InstrTissRec-MTAM).

### Evaluation

During validation and testing, we uniformly sample fixed-length sub-clips, e.g., 16 frames, from each full video clip. The final video-level prediction is the average of the individual model’s sub-clip scores. For the multi-label datasets (CholecT45-Vid, GraSP-Vid), we compute the average precision (AP) for each class as the area under the precision-recall curve per full video clip. The mean average precision (mAP) is obtained by taking the unweighted mean of the AP scores across all full video clips. The mAP is calculated separately for each subtask as well as for the combined task (triplet or doublet). For single-label datasets (SAR-RARP-Vid), we evaluate performance using the accuracy metric.

We follow the official train-test splits for all dataset [1, 15, 16, 24].

### Results

In this section, we first compare the performance of image-based and spatio-temporal models to video transformer models. Second, we evaluate the performance of our proposed MTAM. The results are visualized in Table 1. Furthermore, we conduct a computational comparison with results shown in Table 2.

When comparing image-based models, the Swin-S transformer model consistently outperforms the Res50 model across all three datasets. For instance, on CholecT45-Vid, Swin-S achieves a triplet mAP of $30.0 \%$, compared to $23.9 \%$ for Res50. When incorporating a LSTM layer as a temporal model, the performance remains largely unchanged, with only minor improvements observed in certain datasets, e.g., Swin+LSTM in GraSP-Vid and Res50+LSTM in SAR-RARP-Vid. Using a Transformer as a temporal model, improvements are achieved when Res50 is used as a feature extractor.

To compare with previous methods, we train and evaluate two state-of-the-art methods for surgical triplet recognition on the CholecT45-Vid dataset, i.e., Rendevouz and MT4MTL-KD. While Rendevouz shows inferior performance compared to other image-based model, MT4MTL-KD improves the triplet recognition performance compared to Res50. Video vision transformer models demonstrate superior performance over both image-only and spatio-temporal models. The baseline video model, Swin3D-S, outperforms the best non-video models across all datasets, e.g., with relative improvements of $11.1 \%$ and $35.4\%$ over Swin-S on CholecT45-Vid and GraSP-Vid, respectively. Significant improvements can also be shown on the single-label dataset. On the SAR-RARP-Vid dataset, accuracy improved by $16.8\%$ from image-based Swin-S to video-based Swin3D-S. MViTv2 delivers even stronger performance, increasing accuracy by 5.9$\%$ compared to Swin3D-S.

**Fig. 3:**
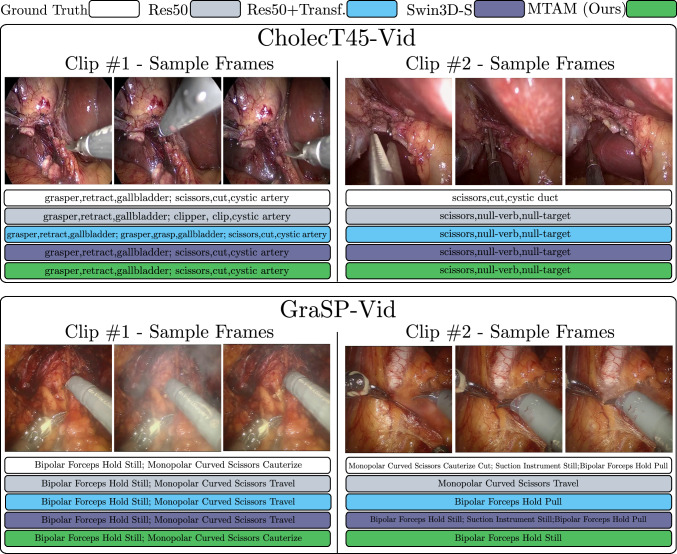
Qualitative results of various methods examined in this study based on the CholecT45-Vid and GraSP-Vid datasets. For each dataset, Clip$\#$1 and Clip$\#$2 indicate correct and challenging examples, respectively. The image-based method (Res50) in Clip$\#$1 (CholecT45-Vid) confuses two visually similar surgical activities: scissors, cut, cystic-artery and clipper, clip, cystic-artery. Methods that incorporate temporal context recognize this activity more accurately. Clip$\#$2 of CholecT45-Vid is 17 s long. None of the methods examined is able to correctly recognize the short-term activity in this clip

Our proposed MTAM exhibits superior performance compared to the Swin3D-S video transformer baseline for surgical instrument-tissue interaction recognition across both CholecT45-Vid and GraSP-Vid datasets. Although little to no improvement is visible in subtask performance for instrument recognition, improvements can be identified for the subtask action and target recognition when comparing MTAM to the Swin3D-S video baseline. Figure 3 visualizes examples of both correct and challenging predictions for the CholecT45-Vid and GraSP-Vid dataset.

The computational analysis visualized in Table 2 shows that video-based models, such as MViTv2 and our proposed MTAM, achieve the highest mAP scores but are also the most computationally demanding, requiring over 64 G FLOPS. In contrast, the MT4MTL-KD model presents a different profile, obtaining a competitive mAP of 28.3 with the lowest resource usage among all architectures at 1.8G FLOPS and 11.7M parameters. This efficiency translates to the lowest inference latency of 4.3ms per clip.

**Fig. 4:**
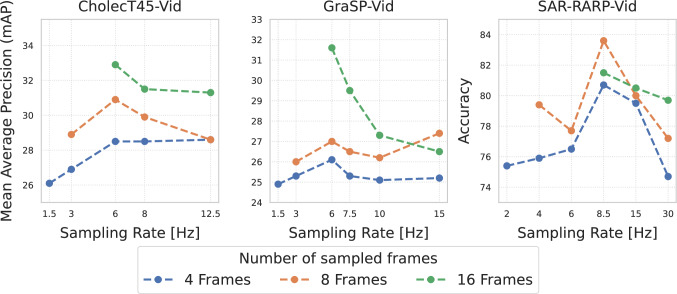
Investigation of the influence of the number of sampled frames and the sampling rate in Hz on the instrument-tissue interaction recognition performance for different datasets

### Ablation studies

In this subsection, we investigate the impact of different temporal sampling rates and the number of sampled frames on the task of instrument–tissue interaction recognition. As shown in Figure 4, increasing the number of sampled frames provided as input to the video transformer leads to improved performance. An analysis across various temporal sampling rates (in Hz) reveals that sampling rates in the range of 6–8.5 Hz yield optimal results across all datasets considered in this study. The optimal sampling parameters were determined to be 16 sampled frames at 6 Hz for CholecT45-Vid and GraSP-Vid, and 8 sampled frames at 8.5 Hz for SAR-RARP50-Vid. In contrast, sampling at lower frequencies (<3 Hz) results in a noticeable performance drop. Also, sampling with higher frequencies (>10 Hz) leads generally to a performance drop. It is important to note that configurations involving very low sampling rates in combination with a high number of sampled frames are infeasible due to the limited duration of the video clips.

We further conduct an ablation study to evaluate the contribution of individual components within the proposed MTAM. Results, presented in Table 3, are obtained on the CholecT45-Vid dataset using test split 4, and are reported as the mean and standard deviation over three different random seeds. Starting from the baseline Swin3D-S model, we first introduce the subtask classifiers used for multi-task learning, which improves performance from 31.6 $\%$ to 32.9$\%$. Subsequently, the integration of the Multi-Task-Attention mechanism and the gating module further boosts performance to 34.2 $\%$ and 35.2 $\%$, respectively.

Additionally, we assess the impact of individual subtask classifiers when used in combination with our proposed MTAM. As shown in Table 4, a gradual addition of subtask classifiers reveals that the inclusion of instrument and target recognition subtasks yields the most significant performance gains. Conversely, using only the action recognition subtask does not result in any improvement in overall performance. The incorporation of multiple subtasks further enhances the model’s efficacy.

**Table 3:** Ablation on the main components of the proposed MTAM. Results are obtained on test-split 4 (CholecT45-Vid)

Method	mAP
Swin3D-S	31.6 ± 0.65
+ Subtask classifier	32.9 ± 0.45
+ Multi-task-attention	34.2 ± 2.01
+ Gating (Ours)	**35.2 ± 0.85**

**Table 4:** Ablation on the influence of the various subtasks on the overall instrument-tissue interaction recognition performance. Results are obtained on test-split 4 (CholecT45-Vid)

Method	mAP
Swin3D-S	31.6 ± 0.65
+ Instrument classifier	32.9 ± 0.31
+ Action classifier	31.5 ± 0.85
+ Target classifier	33.3 ± 1.31
+ Instr & action classifier	32.2 ± 0.91
+ Instr & target classifier	33.9 ± 2.05
+ Action & target classifier	34.4 ± 0.95
+ All subtask classifier	**35.2 ± 0.85**

## Discussion

Prior studies in surgical instrument-tissue interaction recognition predominantly utilize static images or a coarse temporal context for prediction. In this study, we compare image-based, spatio-temporal, and video transformer models, specifically demonstrating the advantage of a fine-grained temporal context for instrument-tissue interaction recognition. Experiments show that image-based Swin-S consistently outperforms ResNet50, reflecting transformers’ ability to capture global dependencies, as shown in work by Gui et al. [6]. Furthermore, proposed methods for surgical triplet recognition, such as Rendevouz, show only limited performance. However, this may be due to the smaller ResNet18 backbone. In contrast, the knowledge distillation approach of MT4MTL-KD achieves a strong mAP score of 28.3, which can compete with more complex models. While adding a decoupled temporal module generally shows suboptimal, the addition of a Transformer to ResNet50 notably improves performance (23.9$\%$ to 27.1$\%$ mAP), in contrast to an LSTM. Dedicated video transformers, such as Swin3D-S or MViTv2, achieve significantly superior performance across all datasets. This highlights the advantage of models designed to learn spatiotemporal information within their architecture for fine-grained surgical instrument-tissue recognition. In our ablation studies concerning the sampling rate, we show that a low sampling rate < 3 Hz or too high > 10 Hz leads to a performance degradation across all datasets. This indicates that too few frames do not capture essential temporal dynamics, while too many introduce redundant information. We identify a sampling rate of 6-8 Hz to be most efficient to capture fine-grained surgical dynamics. This finding provides guidance for future dataset creation and model design in the area of fine-grained surgical instrument-tissue recognition.

Additionally, we propose a Multi-Task-Attention Module, which extends established mixed-attention approaches for instrument-tissue interaction recognition [15, 16]. This module facilitates communication between individual spatio-temporal subtask embeddings through cross-attention and a gating mechanism. Our experiments clearly demonstrate that this approach leads to increased overall performance across all datasets.

The enhanced accuracy of video models corresponds with increased computational demands. High-performing models such as MViTv2 and our MTAM require more computational resources, posing a challenge for real-time clinical applications. In contrast, lightweight architectures like MT4MTL-KD show competitive performance at a fraction of the computational cost and a significantly lower latency, making them more suitable for resource-constrained environments.

In summary, our work demonstrates the superiority of feature extraction via video transformer models over image-based methods. This highlights a promising direction for downstream tasks such as temporal action localization, where robust features are crucial for the precise localization of activities in untrimmed surgical videos. Despite these advancements, our work does not address the high class imbalance present in the data sets examined, nor did we explore optimization techniques to reduce the inference time of our best-performing model. This remains a promising and necessary step to further improve performance and facilitate the clinical translation of surgical instrument-tissue recognition systems.

## Conclusion

In this paper, we demonstrate, through comprehensive experiments, the benefits of leveraging fine-grained temporal context for the recognition of surgical instrument-tissue interactions, an aspect that was largely underexplored in prior studies. Furthermore, we propose a MTAM, which leverages cross-attention to enhance communication between distinct subtasks, thereby further improving surgical instrument-tissue interaction recognition performance. For future work, further approaches to address class imbalance or adapting feature extraction using video transformers for the task of activity localization in untrimmed surgical videos can be investigated.
